# Transcriptomic and Metabolomic Analysis Reveals Multifaceted Impact of Gcn5 Knockdown in *Drosophila* Development

**DOI:** 10.3390/metabo14120680

**Published:** 2024-12-04

**Authors:** Youfeng Li, Yue Xu, Ruike Li, Sirui Huang, Qiong Wu, Jing Yan, Zhigang Jiang, Xiushan Wu, Fang Li, Yuequn Wang, Yongqing Li, Xiongwei Fan, Wuzhou Yuan

**Affiliations:** The Laboratory of Heart Development Research, College of Life Science, Hunan Normal University, Changsha 410081, China; 202010140215@hunnu.edu.cn (Y.L.); xuyue@hunnu.edu.cn (Y.X.);

**Keywords:** Gcn5, knockdown, transcriptome, metabolome, *Drosophila*, pupation, cardiac physiology

## Abstract

**Background:** General control nonderepressible 5 (Gcn5) is a lysine acetyltransferase (KAT) that is evolutionarily conserved across eukaryotes, with two homologs (Kat2a and Kat2b) identified in humans and one (Gcn5) in *Drosophila*. Gcn5 contains a P300/CBP-associated factor (PCAF) domain, a Gcn5-N-acetyltransferase (GNAT) domain, and a Bromodomain, allowing it to regulate gene expression through the acetylation of both histone and non-histone proteins. In *Drosophila*, Gcn5 is crucial for embryonic development, with maternal Gcn5 supporting early development. However, the functional mechanisms of Gcn5 after the depletion of maternal deposits remain unclear. **Methods:** Our study employed the Gal4/UAS-RNAi system to achieve whole-body or heart-specific *Gcn5* knockdown in *Drosophila* and selected 96-hour-old surviving larvae for transcriptomic and metabolomic analyses. **Results:** Omics results revealed that Gcn5 knockdown significantly impacts various metabolic pathways, as well as lysosomes, non-homologous end-joining, Toll and Imd signaling pathways, and circadian rhythms, among others. Furthermore, defects in chitin synthesis may be associated with impaired pupation. Additionally, heart-specific *Gcn5* knockdown affected cardiac physiology but appeared to have a potential protective effect against age-related cardiac decline. **Conclusions:** These findings deepen our understanding of Gcn5’s roles in *Drosophila* development and provide valuable insights for developing Gcn5-targeted therapies, particularly considering its involvement in various human diseases.

## 1. Introduction

General control nonderepressible 5 (Gcn5) is the first lysine acetyltransferase (KAT) identified and cloned in yeast, and it is evolutionarily conserved across eukaryotes [1,2]. In humans, there are two Gcn5 homologs, Kat2a and Kat2b, while *Drosophila* possesses a single homolog, Gcn5 [3]. Gcn5 shares the domains that are common to all Gcn5 homologs, including an N-terminal P300/CBP-associated factor (PCAF) domain, a C-terminal Bromodomain, and a Gcn5-N-acetyltransferase (GNAT) domain [2]. The PCAF domain is primarily involved in nucleosome recognition, while the GNAT domain functions as an acetyltransferase, transferring the acetyl group from acetyl-CoA to the ε-amino group of internal lysine residues [4]. The Bromodomain is responsible for recognizing histone acetyl groups and the recruitment of other factors necessary for transcription [5,6,7].

Gcn5 was initially identified as a histone acetyltransferase; however, thousands of non-histone substrates have been described using high-throughput approaches in organisms ranging from bacteria to humans in recent years [8,9]. As a key enzyme in epigenetics, Gcn5 regulates various signaling pathways by modulating the acetylation levels of histones, non-histones, and numerous transcription factors [10]. In humans, Gcn5-mediated acetylation is associated with chromatin remodeling, autophagy, neuronal apoptosis, cell proliferation, differentiation, DNA repair, and inflammation, and regulates cell energetic state and lipid metabolism [10,11,12]. Gcn5 has been extensively studied for its role in epigenetic regulation and its involvement in the pathogenesis of various diseases, including cancers, diabetes, obesity, metabolic disease and syndrome, dyslipidemia, neurological disorders, infectious diseases, immune disorders, heart failure, muscle diseases, and aging [10,11]. Notably, the inhibition of histone acetyltransferase Gcn5 has been shown to extend lifespan in both yeast and human cell lines [13]. As a result, there is significant interest from both academic researchers and pharmaceutical companies in developing Gcn5 modulators, leading to the discovery of numerous small molecular inhibitors [10,14]. Unfortunately, these inhibitors come with significant limitations, with current candidates like DC_HG24-01 [15], DC_G16-11 [16], and GSK699 [17] still in the preclinical stage and not yet approved for clinical use.

In *Drosophila*, null *Gcn5* alleles obstruct the initiation of both oogenesis and metamorphosis, whereas hypomorphic *Gcn5* alleles impair the formation of adult appendages and cuticles [18]. Notably, a significant amount of Gcn5 protein is detected in oocytes and presyncytial embryos in *Drosophila*, and this maternal contribution of Gcn5 may be sufficient to facilitate embryonic and larval development [18,19]. Maternal effects could potentially interfere with investigations into the function and mechanism of Gcn5; therefore, it is crucial to differentiate between maternal and non-maternal effects when studying Gcn5. Recent research has successfully achieved the knockdown of maternal *Gcn5* using a germline-specific Gal4 driver, demonstrating that maternally deposited Gcn5 is crucial for embryonic development. The cellularization rate (reaching the cellularization stage) of maternal *Gcn5* knockdown *Drosophila* embryos is approximately 25%, with a postcellularization lethality rate (PCLR) of 84.5%. RNA sequencing (RNA-seq) experiments have revealed that maternal Gcn5 is responsible for the transcription of several housekeeping genes and genes involved in postembryonic development [19]. However, there are few studies on the functional mechanisms of Gcn5 after the maternal deposit is depleted. Further research will help in understanding the pathogenesis of Gcn5-related diseases and guide drug development.

In humans, Gcn5 has been extensively studied for its role in the epigenetic regulation and pathogenesis of various cancers and diseases [10]. However, the OMIM (Online Mendelian Inheritance in Man) database has not yet recorded any genetic diseases associated with Gcn5, suggesting that Gcn5 may be involved in the early regulatory processes of biological development leading to early developmental lethality, or may play a role in regulating disease processes through epigenetic modification. Given that whole-body *Gcn5* knockdown is lethal, the application of tissue-specific knockdown will facilitate a more in-depth exploration of Gcn5 in various diseases. Over the past decade, multiple studies have shown that various epigenetic enzymes, such as lysine acetyltransferases (KATs), play important roles in controlling changes in gene expression in heart disease [20]. A recent study found that Kat2a is involved in the intracellular lipid accumulation observed in cardiac mesenchymal stromal cells in patients with arrhythmogenic cardiomyopathy (ACM) [21]. Additionally, another study identified Kat2b as a susceptibility gene for kidney and heart disease in ADD3-associated disorders [22]. Gcn5 could be a novel pharmacological target for heart disease.

In this study, we generated a whole-body knockdown of *Gcn5* using the Gal4/UAS-RNA interference (RNAi) system with a ubiquitous driver (da-Gal4) in *Drosophila*. To minimize maternal interference, we selected 96 h larvae for transcriptomic and metabolomic analyses. Our findings indicate that whole-body *Gcn5* knockdown primarily impacts various metabolic pathways, lysosomes, non-homologous end-joining, Toll and Imd signaling pathways, the circadian rhythm of flies, neuroactive ligand–receptor interactions, and ABC transporters. Core gene analysis suggests that abnormalities in chitin may be a key factor preventing pupation in *Drosophila* with whole-body *Gcn5* knockdown. Additionally, we observed that heart-specific *Gcn5* knockdown disrupts normal cardiac physiology in *Drosophila*; however, as the organism ages, Gcn5 knockdown appears to have a protective effect on cardiac aging. In summary, our study deepens the understanding of Gcn5’s role in *Drosophila* development through transcriptomic and metabolomic approaches, offering new insights and potential strategies for the future development of Gcn5-related therapeutics.

## 2. Materials and Methods

### 2.1. Drosophila Husbandry

*Drosophila* stocks were cultured in standard media at 25 °C with 60% humidity in a 12 h light and 12 h dark cycle. The ubiquitous driver da-GAL4 and heart-specific driver Hand-Gal4 were kindly provided by Ranhui Duan and Min Tang, respectively. The UAS-Gcn5-RNAi line and the Background line (y v; attP2, y+) were obtained from Tsinghua Fly Center (thanks to Dr. Norbert Perrimon, at Harvard Medical School, and Dr. Jian-Quan Ni at Tsinghua Fly Center, School of Medicine, Tsinghua University). The UAS-Gcn5-RNAi line and the Background line were crossed to da-GAL4 or Hand-Gal4 flies, respectively, and incubated at room temperature throughout development. The fruit flies were anesthetized by directing a small stream of carbon dioxide (CO_2_) gas into the culture tube containing the flies. After a brief exposure to CO_2_, the flies were carefully transferred to a plate, where a continuous flow of CO_2_ was maintained to keep the flies in an anesthetized state for the duration of the experiment. Briefly, da-GAL4 or Hand-Gal4 virgin flies were selected and crossed with UAS-Gcn5-RNAi line male flies, respectively, to obtain whole-body *Gcn5* knockdown and heart-specific *Gcn5* knockdown flies.

### 2.2. Collection of 96 h Larvae

Prepare thirty virgin female fruit flies and fifty 3- to 5-day-old male fruit flies and evenly distribute them into three separate culture tubes. Every 4 h, transfer the flies to a new food tube to initiate a new round of experimental timing. After 96 h, carefully transfer the culture medium containing the larvae to an empty Petri dish. Manually collect the surviving larvae under a stereomicroscope, quickly freeze them in liquid nitrogen, and then store them at −80 degrees Celsius, or proceed with transcriptome and metabolome experiments.

### 2.3. Transcriptome and Metabolome Data Analysis

Transcriptome and metabolome analyses were performed by the SHANGHAI BIOPROFILE Company. The transcriptome and metabolome analyses were performed using three biological replicates. Each replicate contained at least 20 whole-body *Gcn5* knockdown or control larvae.

Transcriptome analysis: Total RNA was extracted from the tissue using TRIzol Reagent according to the manufacturer’s instructions. Only high-quality RNA samples (OD260/280 = 1.8~2.2, OD260/230 ≥ 2.0, >1 μg) were used to construct the sequencing library. The samples underwent Next-Generation Sequencing (NGS) on the Illumina sequencing platform. The libraries were subjected to paired-end (PE) sequencing. Differentially expressed genes (DEGs) were evaluated through DESeq, with screening conditions set at a significance threshold of |log2FC| > 1 and *p* < 0.05. GO and KEGG pathway enrichment analyses were performed using David v2022 (https://david.ncifcrf.gov/, accessed on 19 August 2024), with *p* < 0.05 as the cut-off criteria. PPI networks were developed using STRING version 11.0b (https://string-db.org/, accessed on 19 August 2024) alongside Cytoscape (version 3.10.1). Heat maps were plotted by the website (https://www.bioinformatics.com.cn, accessed on 26 September 2024), a free online platform for data analysis and visualization.

Metabolome analysis: Before the extraction of metabolites, samples were weighed, and the dried lyophilized material was ground in a 2 mL Eppendorf tube containing a 5 mm tungsten bead for 1 min at 65 Hz in a Grinding Mill. Metabolites were extracted using 1 mL precooled mixtures of methanol, acetonitrile, and water (*v*/*v*/*v*, 2:2:1) and then placed in ice baths for 1 h ultrasonic shaking. Subsequently, the mixture was placed at −20 °C for 1 h and centrifuged at 14,000× *g* for 20 min at 4 °C. The supernatants were recovered and concentrated to dryness in vacuum conditions. Metabolomics profiling was performed with a UPLC-ESI-Q-Orbitrap-MS system (UHPLC, Shimadzu Nexera X2 LC-30AD, Shimadzu, Japan) paired with Q-Exactive Plus (Thermo Scientific, San Jose, CA, USA). Models were built on principal component analysis (PCA) and partial least-square discriminant analysis (OPLS-DA). OPLS-DA allowed the determination of discriminating metabolites using the variable importance on projection (VIP). The VIP score value indicates the contribution of a variable to the discrimination between all the classes of samples. The mean VIP value is 1, and usually, VIP values over 1 are considered significant. To identify the perturbed biological pathways, the differential metabolite data underwent KEGG pathway analysis using the KEGG database (http://www.kegg.jp, accessed on 19 August 2024). Enriched KEGG pathways were nominally statistically significant at the *p* < 0.05 level.

### 2.4. Semi-Intact Drosophila Heart Preparation and Cardiac Function Analysis

*Drosophila* semi-intact hearts were prepared as previously described [23,24]. Videos of beating hearts were recorded for 30 s with a high-speed EM-CCD camera (Hamamatsu; Shizuoka; Japan 110 fps/s) at a speed of 110 images/s. Data acquisition was performed using HC Image software (Hamamatsu, Version 2.1.1.0). Videos were analyzed with Semi-automatic Optical Heartbeat Analysis software (SOHA, Version 3.4.0.0, provided by Ocorr and Bodmer) to quantify heart periods, systolic and diastolic intervals, systolic and diastolic diameters, arrhythmia indexes, and fractional shortening and to produce M-mode records.

### 2.5. Behavioral Trajectory Tracking Analysis

Anesthetize the 5-week-old heart-specific *Gcn5* knockdown adult flies by directing a small stream of carbon dioxide (CO_2_) gas into the culture tubes. Then, promptly transfer them to a 48-well Petri dish, ensuring that each well contains a single fruit fly, and then secure the lid. Leave at room temperature for 2 h to ensure that the fruit flies fully recover from anesthesia. Then, put it into the zebrafish behavioral observation box (France/ViewPoint/ZebraBox), open the “ViewPoint Application Manager” behavior analysis software (Version 5.18.0.0), and select the “behavior tracking” experiment type. Set thresholds for low, medium, and high speeds based on the speed of fruit fly movement: low-speed movement (<1 cm/s); medium-speed movement (>1 cm/s and <2 cm/s); high-speed movement (>2 cm/s). Monitor fly movement in real time for 1 h, and during this, record the movement trajectory and the total distance traveled at each speed for subsequent analysis.

### 2.6. Lifespan Assay

On the day of eclosion, adult flies were gathered and kept in groups of 25 per tube at a temperature of 25 °C, with a humidity level of 60% and a light/dark cycle of 12 h each. Every 3 days, the flies were moved to fresh tubes, and their survival rate was recorded. A log-rank (Mantel−Cox) test was used to compare the lifespan among different genotypes. Two replicates (about 100 flies in each genotype in every replicate) were used in each test.

### 2.7. Western Blot

Protein extracts were prepared following established protocols, and the proteins underwent separation and blotting using SDS-PAGE. The following antibodies were used: Acetyl-Histone H3 (Lys9) Rabbit Polyclonal Antibody (beyotime, Shanghai, China, AF5611, 1:1000 dilution); Histone H4 Rabbit Polyclonal Antibody (beyotime, Shanghai, China, AF7107, 1:1000 dilution); and antibody to GAPDH (Sigma-Aldrich, Burlington, MA, USA, 1:2000 dilution). Antibody binding was detected using a ChemiDocTMXRS+system (Bio-rad, Shanghai, China).

### 2.8. Data Analysis and Visualization

The data were statistically analyzed using GraphPad Prism 8.0 software and subsequently graphed. An unpaired *t*-test was used to assess the differences between groups. Data are presented as means ± SEM from at least three replicates, where ns *p* > 0.05, * *p* < 0.05, ** *p* < 0.01, and *** *p* < 0.001.

## 3. Results

### 3.1. Gcn5 Is Essential for Drosophila Metamorphosis

**Figure 1:**
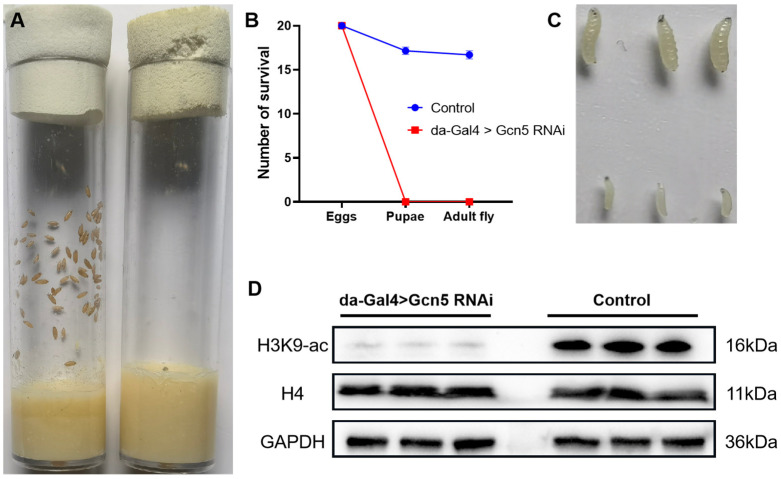
Whole-body knockdown of *Gcn5* in *Drosophila*. (**A**) Fly embryos with whole-body knockdown of *Gcn5* were unable to pupate. Left: control group (da-Gal4>y v; attP2, y+); right: experimental group (da-Gal4>Gcn5 RNAi). (**B**) The graph shows the survival number of *Drosophila* at two different developmental stages when eggs were transferred to a new medium. Data analysis was performed with 20 eggs per tube and repeated 10 times. The whole-body knockdown of *Gcn5* prevents eggs from developing into pupae. Data are displayed as mean ± SEM. (**C**) The 96 h larvae of whole-body *Gcn5* knockdown *Drosophila* were severely reduced in size. Up: control group (da-Gal4>y v; attP2, y+); down: experimental group (da-Gal4>Gcn5 RNAi). (**D**) Western blot analysis of Histone H4 and Histone H3 lysine 9 acetylation (H3K9-ac) in 96 h larvae.

We generated a whole-body knockdown strain of *Gcn5* using the Gal4/UAS-RNA interference (RNAi) system with a ubiquitous driver (da-Gal4) in *Drosophila* (Figure S1A). Our findings revealed that, compared with the control group, the fly embryos with a whole-body knockdown of Gcn5 were unable to pupate (Figure 1A,B). The previous literature has reported that Gcn5 exhibits maternal effects, suggesting that maternally deposited Gcn5 may be sufficient to support embryonic and larval development [18,19].

In order to avoid the influence of maternal Gcn5, we tried to select flies that could develop to the latest possible stage before lethality for our experiments. We observed that a small number of whole-body *Gcn5* knockdown *Drosophila* embryos survived to 96 h at room temperature; so, these 96 h larvae were selected for subsequent experiments. Compared with the control group, the 96 h larvae of whole-body *Gcn5* knockdown *Drosophila* were severely reduced in size (Figure 1C). Histone H3 is the most well-studied acetylation target of Gcn5. We further tested the acetylation level of Histone H3, and our results demonstrated that whole-body *Gcn5* knockdown indeed significantly reduced the acetylation level of H3K9 (Figure 1D). Thus, Gcn5 is crucial for the metamorphic development of *Drosophila*.

### 3.2. Transcriptome Analysis

Research has shown that Gcn5 acts as both histone and non-histone lysine acetyltransferase [8,9]. As a histone acetyltransferase, Gcn5 plays a pivotal role in shaping the epigenetic landscape and facilitating chromatin modifications that are associated with transcriptional activation. Meanwhile, Gcn5 is capable of acetylating non-histone proteins, such as the tumor suppressor p53 [25] and the c-Myc oncoprotein [26], both of which are involved in regulating gene transcription. So, we explored the effect of Gcn5 on gene expression via poly(A)-selected RNA sequencing (RNA-Seq).

**Figure 2:**
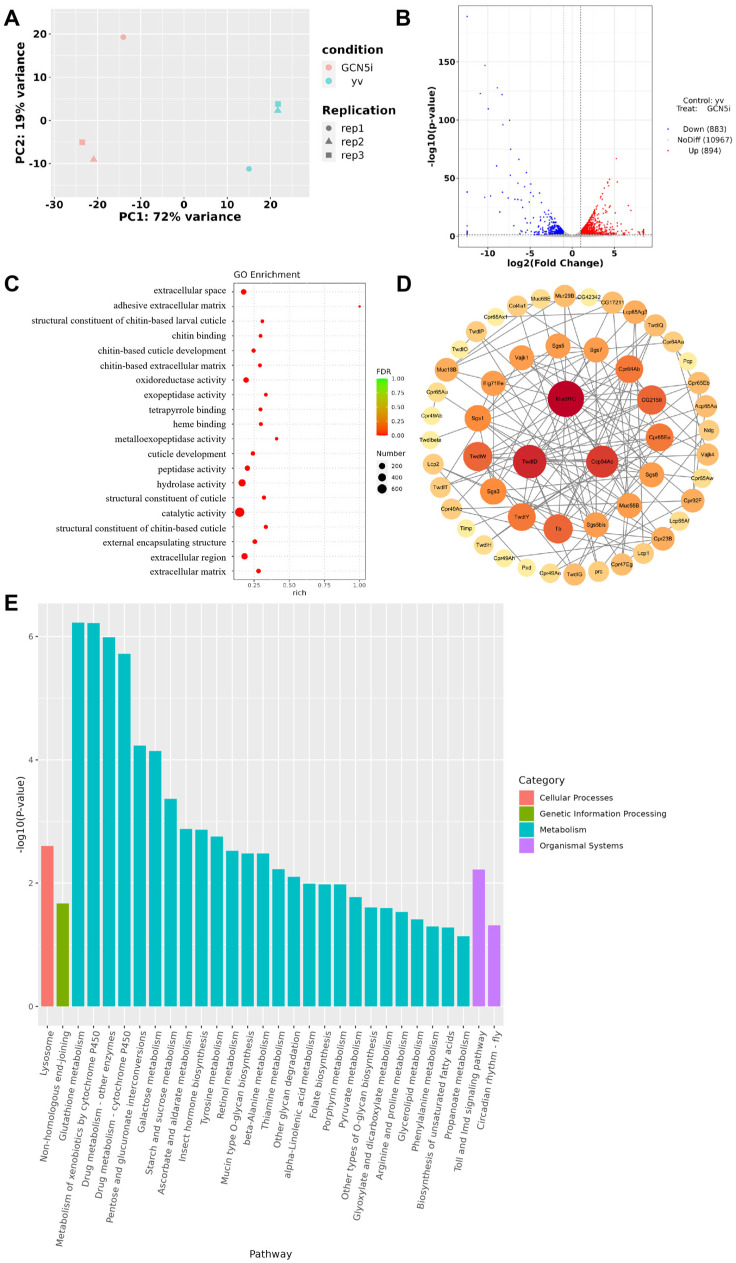
Effects of whole-body knockdown of *Gcn5* on the transcriptome of 96 h larvae. (**A**) Principal component analysis (PCA) of transcriptome data. (**B**) Volcano plot of differentially expressed genes (DEGs). Each dot on the plot represents a single gene; blue dots represent downregulated genes, red dots represent upregulated genes, and gray dots represent genes with no significant changes (significance threshold: |log2FC| > 1.0 and *p* < 0.05). (**C**) Bubble chart of top GO enrichment analysis terms. (**D**) PPI network analysis for external encapsulating structures related to differential genes. (**E**) Bar chart of top 30 KEGG pathway enrichment analysis pathways.

The principal component analysis (PCA) of the mRNA expression data indicated significant separation, with principal component 1 (PC1) contributing 72% and principal component 2 (PC2) contributing 19%, together explaining a total variance of 91% (Figure 2A). Compared with the control group, 883 genes were downregulated, and 894 genes were upregulated in whole-body *Gcn5* knockdown flies (fold-change > 2, *p*-value < 0.05) (Figure 2B, Supplementary Table S1). Gene Ontology (GO) analysis revealed that their roles were mainly involved in catalytic activity, hydrolase activity, peptidase activity, oxidoreductase activity, exopeptidase activity, extracellular space, extracellular region, external encapsulating structure, cuticle development, chitin-based cuticle development, chitin-based extracellular matrix, structural constituent of chitin-based cuticles, and chitin binding (Figure 2C).

Our findings indicate that, in addition to its catalytic activity, Gcn5 significantly influences the external encapsulating structure, cuticle development, and the extracellular region. This may be the reason why whole-body *Gcn5* knockdown *Drosophila* cannot pupate normally. We conducted a core gene screening of the differentially expressed genes within the relevant Gene Ontology (GO) term for external encapsulating structures, identifying three core genes, Muc91C, TwdlD, and Ccp84Ac, all of which are associated with chitin-based larval cuticles (Figure 2D). During the development of *Drosophila*, chitin metabolism is critical for morphological changes, helping *Drosophila* complete the transition from larvae to adults. Therefore, abnormalities in chitin metabolism may be a primary reason for the pupation failure observed in *Drosophila* with whole-body *Gcn5* knockdown. KEGG (Kyoto Encyclopedia of Genes and Genomes) pathway enrichment analysis revealed that whole-body *Gcn5* knockdown mainly affects metabolic pathways, including glutathione metabolism, metabolism of xenobiotics, drug metabolism, pentose and glucuronate interconversions, galactose metabolism, starch and sucrose metabolism, insect hormone biosynthesis, tyrosine metabolism, etc. Additionally, it is involved in lysosomes, non-homologous end-joining, Toll and Imd signaling pathways, and the circadian rhythms of flies (Figure 2E).

### 3.3. Metabolome Analysis

The acetyltransferase activity of Gcn5 is dependent on its capacity to catalyze the transfer of the acetyl group from acetyl-CoA, a central metabolite in cellular energy metabolism [12,27]. Additionally, Gcn5 is capable of acetylating non-histone proteins, such as PGC-1α, a transcriptional coactivator that regulates genes associated with energy metabolism and mitochondrial biogenesis [28]. Animal models have demonstrated that the expression of Gcn5 is increased by a high-fat diet and decreased by fasting [29,30]. Research has also revealed that Gcn5 functions as an inhibitor of autophagy and lysosomal biogenesis in *Drosophila*, a key process of cell catabolism [31]. Furthermore, our transcriptomic results also show that Gcn5 plays a significant role in metabolic pathways. Collectively, these findings indicate that Gcn5 is a crucial regulator of cellular metabolism. To further validate the results of the transcriptomic analysis, this study examined the effects of whole-body *Gcn5* knockdown on metabolites in 96 h larvae using the UPLC-Q-Exactive Plus MS technique.

**Figure 3:**
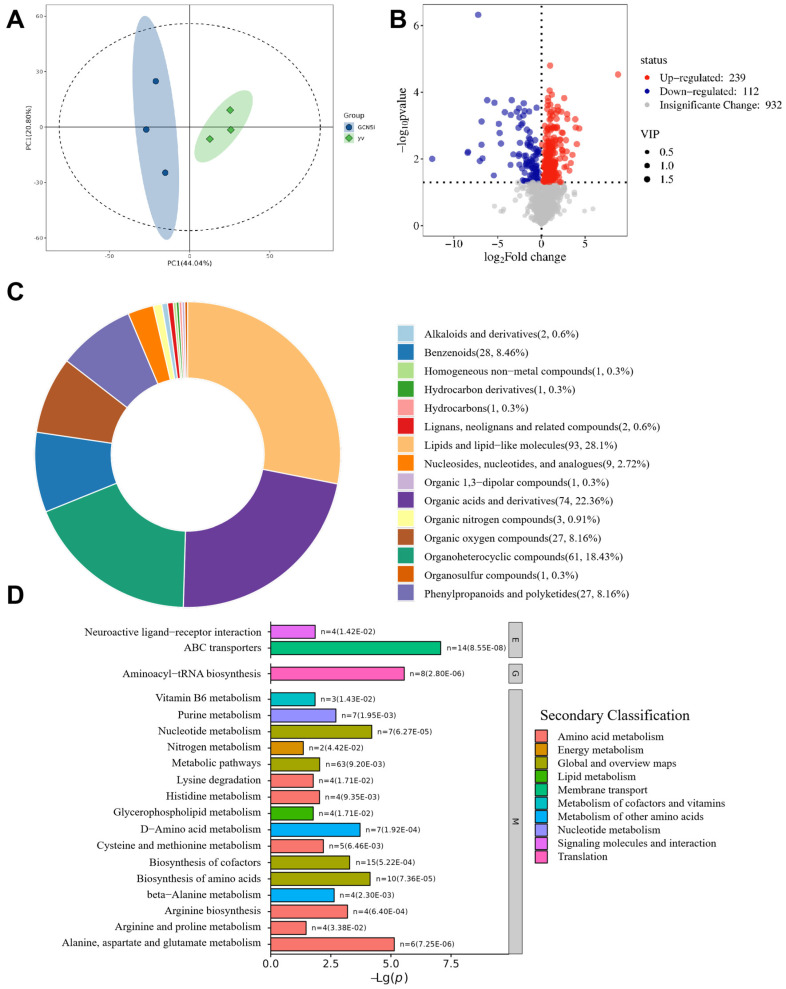
Effects of whole-body knockdown of *Gcn5* on the metabolome of 96 h larvae. (**A**) Principal component analysis (PCA) of metabolome data. (**B**) Volcano plot of differential metabolites. Each dot represents a metabolite, and the dot size represents the VIP value; purple dots represent downregulated metabolites, red represents upregulated metabolites, and gray dots represent metabolites with no significant changes (significance threshold: VIP > 1.0 and *p* < 0.05). (**C**) Ring map of differences in metabolites’ HMDB Super Class classification. (**D**) Bar chart of differences in metabolites’ KEGG pathway enrichment (Level 1 pathway classification: M: metabolism; E: environmental information processing; G: genetic information processing).

The principal component analysis (PCA) of the metabolite content showed significant separation, with 20.80% contribution from principal component 1 (PC1) and 44.04% contribution from principal component 2(PC2), with an overall contribution of 64.84% (Figure 3A). The OPLS-DA model established with this experimental data has R2 and Q2 ≥ 0.5. The model is stable and reliable, and the model has good explanatory and predictive capabilities. With VIP > 1.0 and *p* < 0.05 as the threshold for significance, a total of 351 significantly differential metabolites, including 239 increased and 112 decreased metabolites (Figure 3B, Supplementary Table S2), were determined. Expression change analysis and functional pathway analysis were further conducted. According to the structure and function of the metabolites, the differential metabolites were classified according to the Super Class of HMDB, mainly including lipids and lipid-like molecules (93, 28.1%), organic acids and derivatives (74, 22.36%), organoheterocyclic compounds (61, 18.43%), benzenoids (28, 8.46%), phenylpropanoids and polyketides (27, 8.16%), organic oxygen compounds (27, 8.16%), nucleosides, nucleotides, and analogs (9, 2.72%), etc. (Figure 3C). The KEGG enrichment analysis indicated that the differential metabolites were primarily enriched in several metabolic pathways, including alanine, aspartate, and glutamate metabolism; nucleotide metabolism; biosynthesis of amino acids; D-amino acid metabolism; biosynthesis of cofactors; arginine biosynthesis; and beta-alanine metabolism. Additionally, the analysis encompassed neuroactive ligand–receptor interactions, ABC transporters, and aminoacyl-tRNA biosynthesis (Figure 3D).

### 3.4. Correlation Analysis of Transcriptome and Metabolome

**Figure 4:**
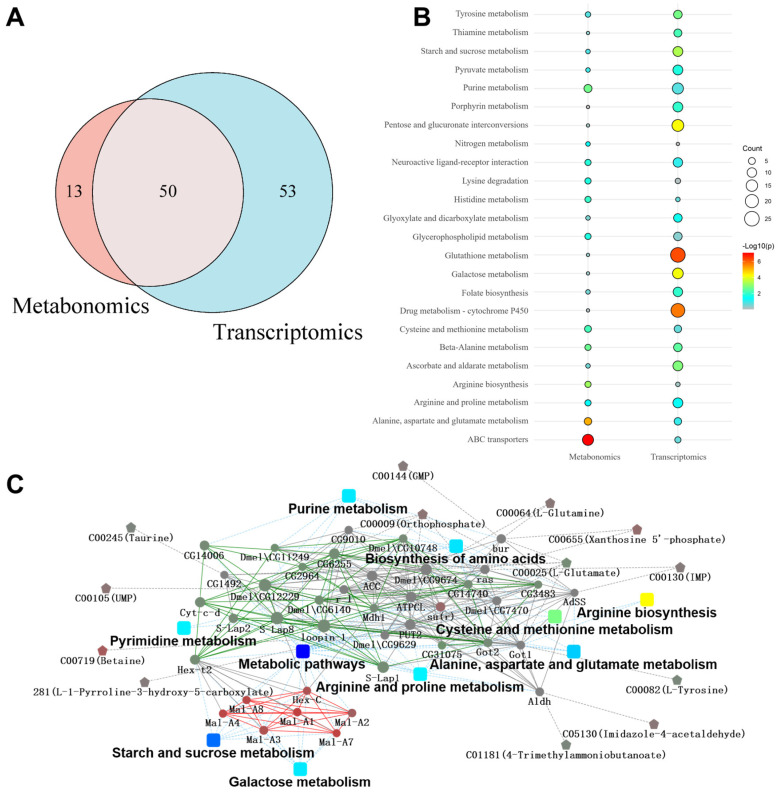
Correlation analysis of transcriptome and metabolome data. (**A**) Venn diagram revealed the common KEGG pathways enriched in both transcriptome and metabolome. (**B**) Metabolomics and transcriptomics share significant KEGG bubble charts. The size of the dots indicates the number of expressed genes or metabolites in the pathways, and the color of the dots represents the *p*-value of the pathway. (**C**) PPI network of combined metabolome and transcriptome analyses. The rectangular nodes in the diagram represent the KEGG pathway (significant *p*-values are represented by a yellow-blue gradient; the darker the blue, the more significant). The circular nodes represent proteins/genes, and the pentagon represents metabolites (red indicates upregulation and green indicates downregulation).

The common KEGG pathways enriched in both the transcriptome and metabolome were analyzed. The Venn diagram illustrating these KEGG pathways indicated that the majority of pathways enriched through metabolomic analysis were also identified in the transcriptomic data (Figure 4A). Commonly recognized significant pathways include tyrosine metabolism, purine metabolism, starch and sucrose metabolism, glycerophospholipid metabolism, arginine and proline metabolism, and alanine, aspartate, and glutamate metabolism, alongside neuroactive ligand–receptor interactions and ABC transporters (Figure 4B). Furthermore, the PPI network was mapped using combined transcriptome and metabolome analysis. A series of metabolites and candidate genes were revealed through a combined analysis of the metabolome and transcriptome (Figure 4C).

Taken together, these data suggest that Gcn5 regulates *Drosophila* growth, metamorphosis, and other developmental processes primarily through metabolic processes.

### 3.5. Gcn5 Is Required to Maintain Normal Heart Physiology

Research has shown that Gcn5 is associated with various cancers and numerous diseases [10,11]. However, the OMIM database has yet to record any genetic diseases associated with Gcn5. Given that the whole-body knockdown of *Gcn5* is lethal, targeting Gcn5 in specific tissues can enhance our understanding of its functional role. Recent reports indicate that Kat2b is a susceptibility gene for heart disease [22], and Kat2a is involved in the intracellular lipid accumulation observed in cardiac mesenchymal stromal cells in patients with arrhythmogenic cardiomyopathy [21]. Our transcriptome and metabolome results show that Gcn5 is widely involved in material and energy metabolism. As a central organ driving vital physiological processes, the heart relies heavily on precise metabolic regulation to maintain its function and overall homeostasis. Consequently, we utilized the Hand-gal4 driver (expressed in myocardial as well as pericardial cells) crossed with Gcn5 RNAi lines to achieve heart-specific knockdown of *Gcn5* in *Drosophila* (Figure S1B). We then tested cardiac function in one-week-old flies (corresponding to human youth) using the semi-automated optical heartbeat analysis (SOHA) system. This technology can analyze and quantify the rhythm and the dynamics of cardiac contraction, including heart period (HP), diastolic interval (DI), systolic interval (SI), diastolic diameter (DD), systolic diameter (SD), fractional shortening (FS, a classic measure of cardiac output), and arrhythmia index (AI). The arrhythmia index (AI) refers to the difference between each heartbeat cycle of the *Drosophila* heart and the median value of the overall heartbeat cycle, reflecting the extent of irregularity in the heartbeats of *Drosophila*.

**Figure 5:**
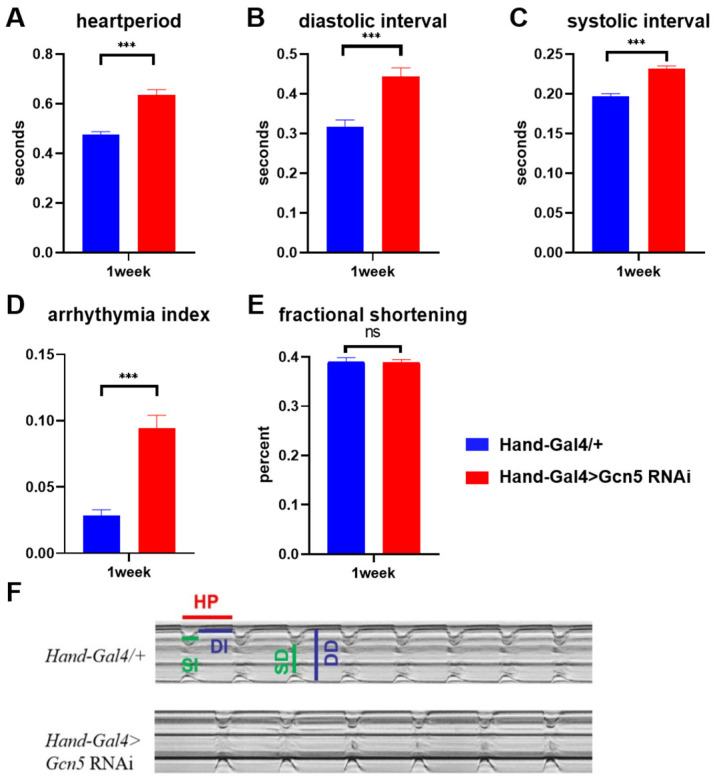
Effect of *Gcn5* knockdown on cardiac function in 1-week-old flies. (**A**) Heart period, (**B**) diastolic interval, (**C**) systolic interval, and (**D**) arrhythmia index are increased in 1-week-old *Gcn5* knockdown flies. (**E**) Fractional shortening is not significantly reduced. (**F**) Representative 5 s M-modes traced from semi-intact *Drosophila* heart preparations reveal the movements of the heart walls (y-axis) over time (x-axis). HP: heart period; SI: systolic interval; DI: diastolic interval; DD: diastolic diameter; SD: systolic diameter; *** *p* < 0.001.

We found that cardiac-specific *Gcn5* knockdown in one-week-old adult flies extended the heart period compared with controls (Figure 5A,F). This was due to increases in both the systolic and diastolic intervals (Figure 5B,C). In addition, the fly heart arrhythmia index increased (Figure 5D), but there was no difference in fractional shortening (Figure 5E). Experimental results showed that *Gcn5* knockdown indeed caused cardiac dysfunction in *Drosophila*.

### 3.6. Heart-Specific Gcn5 Knockdown Makes Aged Flies More Active and Live Longer

Interestingly, we inadvertently observed that heart-specific *Gcn5* knockdown flies are more active at older ages. Consequently, we conducted a further analysis of 5-week-old flies, which correspond to elderly humans.

**Figure 6:**
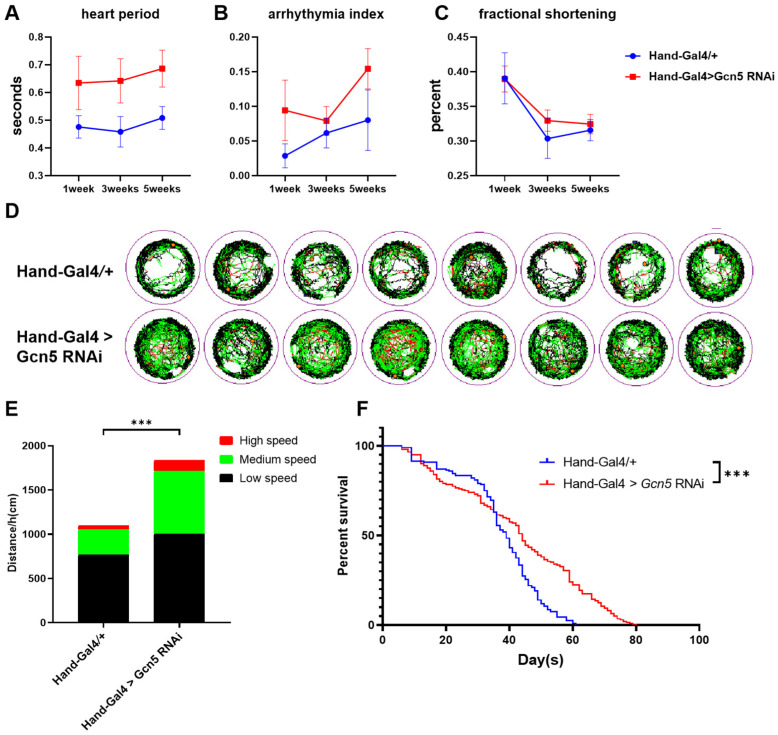
Heart-specific *Gcn5* knockdown plays a protective role in the aging process. (**A**–**C**) Effect of *Gcn5* knockdown on cardiac function with age. (**D**) Movement trajectories of heart-specific *Gcn5* knockdown flies (down) and control flies (up) at 5 weeks of age. Black line: low-speed movement (<1 cm/s); green line: medium-speed movement (>1 cm/s and <2 cm/s); and red line: high-speed movement (>2 cm/s). (**E**) Statistical analysis of the distance traveled by fruit flies in 1 h at various speeds. *** *p* < 0.001 (*n* = 8, unpaired *t*-test). (**F**) Survival curve analysis. Heart-specific *Gcn5* knockdown flies have significantly longer life spans than the control group (*p* < 0.001, Mantel–Cox log-rank test). Graph plots % survival (*n* = 200) versus time (in days) post-eclosion.

We found that the heart period of heart-specific *Gcn5* knockdown flies was prolonged at 1, 3, and 5 weeks (Figure 6A); however, the arrhythmia index decreased and fractional shortening increased at 3 weeks (Figure 6B,C). We further assessed the activity of 5-week-old flies, using a behavior trajectory tracking system to examine the movement patterns of heart-specific *Gcn5* knockdown flies in comparison to wild-type flies over a one-hour period. The experimental results indicated that heart-specific *Gcn5* knockdown flies exhibited significantly increased activity at 5 weeks of age. These flies demonstrated greater overall crawling distances within one hour, with increases observed in low-, medium-, and high-movement distances (Figure 6D,E). A survival curve analysis found that although the mortality rate of heart-specific *Gcn5* knockdown flies before 5 weeks of age was slightly higher, the overall life span of heart-specific *Gcn5* knockdown flies was significantly extended, with a median survival of 44 days compared to 39 days in the control group (Figure 6F). These results suggest that *Gcn5* knockdown may play a protective role in the aging process of the *Drosophila* heart.

## 4. Discussion

As a key epigenetic enzyme, Gcn5 regulates various signaling pathways by modulating the acetylation levels of histones, non-histones, and numerous transcription factors [10]. Since an important stock of Gcn5 protein is detected in oocytes and presyncytial embryos, this maternal contribution of Gcn5 may be sufficient to allow embryonic and larval development. The maternal Gcn5-dependent genes are crucial for later stages of development, as indicated by Gene Ontology (GO) terms associated with postembryonic development and metamorphosis, or they have housekeeping functions [18,19]. However, the mechanisms by which Gcn5 functions after the depletion of the maternal deposit remain to be fully elucidated. To explore this, we generated a whole-body knockdown strain of *Gcn5* using the Gal4/UAS-RNA interference (RNAi) system with a ubiquitous driver (da-Gal4) in *Drosophila*. Our results showed that a small number of whole-body *Gcn5* knockdown *Drosophila* embryos could survive to 96 h at room temperature; however, these larvae are significantly smaller and unable to pupate.

Transcriptome Gene Ontology (GO) analysis indicates that *Gcn5* primarily functions in catalytic activity. Further analysis of the GO results revealed that the failure of whole-body *Gcn5* knockdown Drosophila embryos to pupate is significantly linked to Gcn5’s role in the formation of the external encapsulating structure, cuticle development, and the extracellular region. Insects have evolved a chitin-based cuticle to protect themselves from the environment. Every time the larva undergoes an instar—the developmental phase between successive molts—a new cuticle is synthesized to accommodate the growing body. The *Drosophila* cuticle is a layered structure that includes an envelope layer, a proteinaceous epicuticle, and a procuticle rich in chitin (polymer of N-acetylglucosamine) [32,33]. The chitin microfibrils along with the associated proteins in the procuticle create a distinctive lamella, essential for preserving cuticle tension. Core gene analysis suggests that chitin-based cuticle development abnormalities may be a primary reason why whole-body *Gcn5* knockdown *Drosophila* are unable to pupate.

As one of the most metabolically active organs in the human body, the heart consumes huge amounts of energy through its daily contraction activities. To meet these demands, the heart is able to produce ATP from multiple fuel sources, such as carbohydrates, fats, lactate, amino acids, and ketone bodies. Because of its high metabolic activity, any disruptions in the heart’s metabolic processes are often linked to the development of various cardiovascular diseases. These metabolic disturbances can impair the heart’s ability to efficiently generate energy, contributing to the progression of heart-related conditions [34,35]. Disturbances in cardiac metabolism underlie most cardiovascular diseases. A recent study by Chiara Volani and colleagues found that Gcn5 plays a role in the accumulation of intracellular lipids in cardiac mesenchymal stromal cells from patients diagnosed with arrhythmogenic cardiomyopathy (ACM) [21]. Our metabolomic results suggest that the whole-body knockdown of Gcn5 impacts the metabolism of various amino acids, lipids, and nucleic acids, indicating that Gcn5 may play a crucial role in cardiac development.

In our study, we found that heart-specific *Gcn5* knockdown resulted in abnormal cardiac function during the early stages of development. However, the knockdown of *Gcn5* during cardiac aging seemed to have a protective effect, as older flies exhibited increased activity and an extended lifespan. A study by Peleg et al. demonstrated that the overall level of acetylation increases significantly with age in *Drosophila*, suggesting that elevated acetylation may serve as a hallmark of aging [36]. Additionally, the hyperacetylated proteins identified during aging are involved in metabolic processes, including the final steps of glycolysis, the tricarboxylic acid cycle, and oxidative phosphorylation [36]. More recently, Nan Liu et al. discovered that Gcn5 displays a transcriptional increase in both head and muscle tissues as *Drosophila* ages. The lifespan defects observed in Gcn5 mutants suggest potential modulatory roles of Gcn5 in adult and aging processes [9]. Nan Liu et al. further showed that the age-related increase in acetylation is at least partially attributed to Gcn5 [9]. Thus, Gcn5 may be involved in *Drosophila* heart development in the early stage and also contribute to accelerated heart aging through its role in acetylation during the aging process. However, the mechanisms by which Gcn5 influences early *Drosophila* heart development and its role during aging warrant further in-depth investigations.

Gcn5 plays a crucial role in numerous biological processes. Dysregulation of Gcn5 has been closely linked to various human diseases, especially cancers [37]. Gcn5 modulators hold significant importance for human health and aging. Hence, the exploitation of small molecules targeting Gcn5 is essential for drug design and academic research. Although many small molecular inhibitors have been identified, these compounds have several limitations [10,11]. Currently, there are no Gcn5 inhibitors available for clinical use. Our transcriptomic and metabolomic experiments demonstrate that Gcn5 is extensively involved in cellular metabolic processes. Additionally, the PPI network was mapped using combined transcriptomic and metabolomic analysis. This integrated approach provides an important tool for the mining of metabolic networks and key genes. Our study provides important insights for discovering specific Gcn5 regulators, but further in-depth exploration is necessary. In conclusion, we have constructed a comprehensive map of transcriptomic and metabolomic changes related to Gcn5 during *Drosophila* development and provide valuable data resources for future research.

## Data Availability

All data generated or analyzed during this study are included in this published article and its Supplementary Information Files.

## Supplementary Materials

The following supporting information can be downloaded at https://www.mdpi.com/article/10.3390/metabo14120680/s1: Figure S1: qPCR of *Gcn5* RNA from (A) whole-body *Gcn5* knockdown *Drosophila* pupae and (B) heart-specific *Gcn5* knockdown *Drosophila* hearts; Table S1: Transcriptome data; Table S2: Metabolome data.
