# Supplementary material for: Transcriptomic and Metabolomic Analysis Reveals Multifaceted Impact of Gcn5 Knockdown in Drosophila Development
Source: Metabolites. 2024 Dec 4;14(12):680. doi: 10.3390/metabo14120680 (PMC11678289; doi:10.3390/metabo14120680)
Supplement: Supplementary file 1 [file metabolites-14-00680-s001.zip › Supplementary Figure S1.pdf]

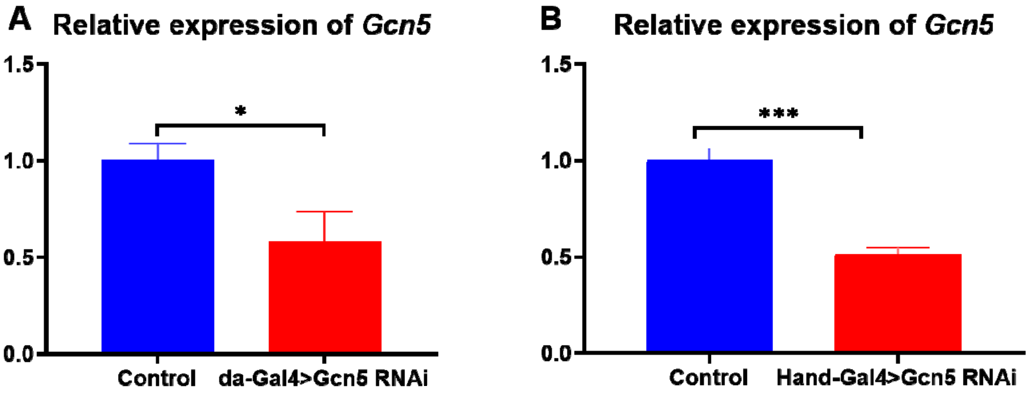

Figure S1. qPCR of Gcn5 RNA from (A)whole-body Gcn5 knockdown Drosophila pupae and (B) heart-specific Gcn5 knockdown Drosophila hearts. Relative expression of Gcn5 was normalized to ribosomal rp49 expression. Significance was determined using a Student's T-test; * $p < 0.05$, *** $p < 0.001$.
