# Supplementary figures and images for: Transcriptomic and Metabolomic Analysis Reveals Multifaceted Impact of Gcn5 Knockdown in Drosophila Development
Source: Metabolites. 2024 Dec 4;14(12):680. doi: 10.3390/metabo14120680 (PMC11678289; doi:10.3390/metabo14120680)

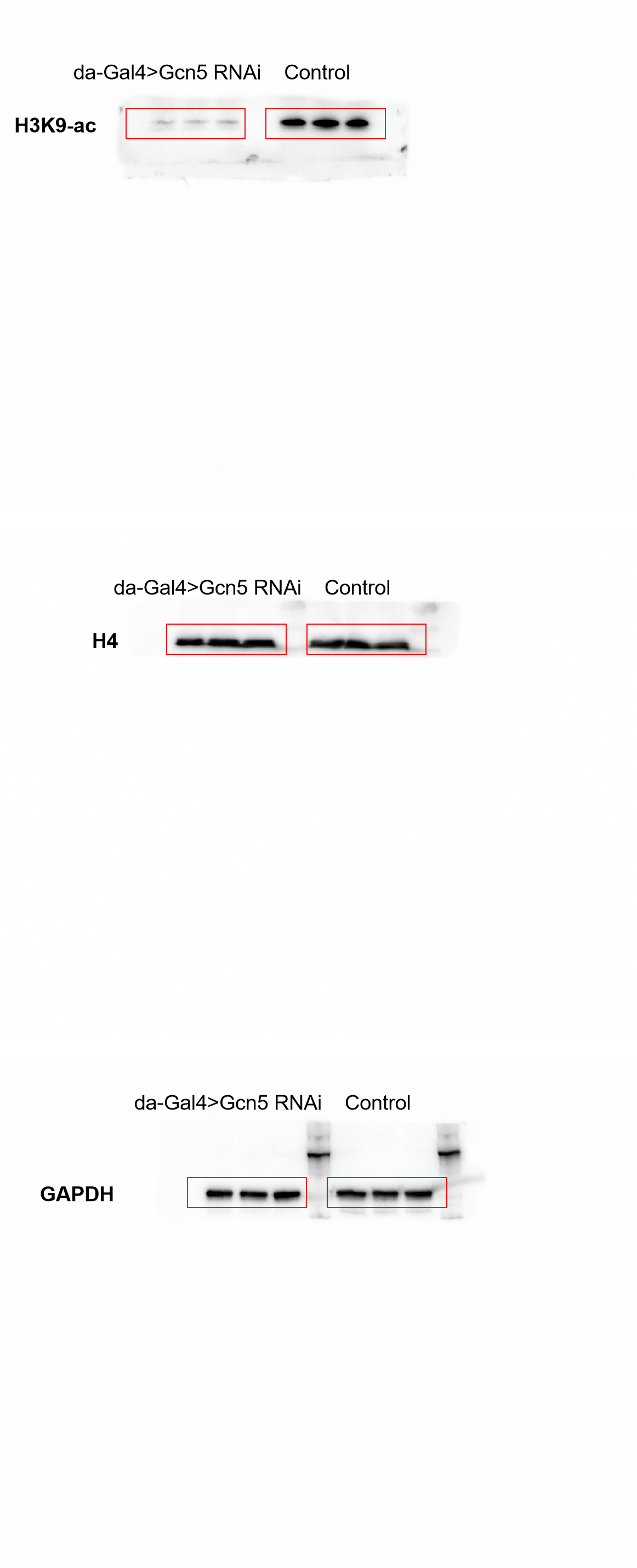

Supplement: Supplementary file 1 [file metabolites-14-00680-s001.zip › WB original Images.png]
